# Supplementary material for: Transhydrogenase and Growth Substrate Influence Lipid Hydrogen Isotope Ratios in Desulfovibrio alaskensis G20
Source: Front Microbiol. 2016 Jun 22;7:918. doi: 10.3389/fmicb.2016.00918 (PMC4916218; doi:10.3389/fmicb.2016.00918)
Supplement: Supplementary file 5 [file Data_Sheet_5.PDF]

Supplemental Figures

Figure S1.

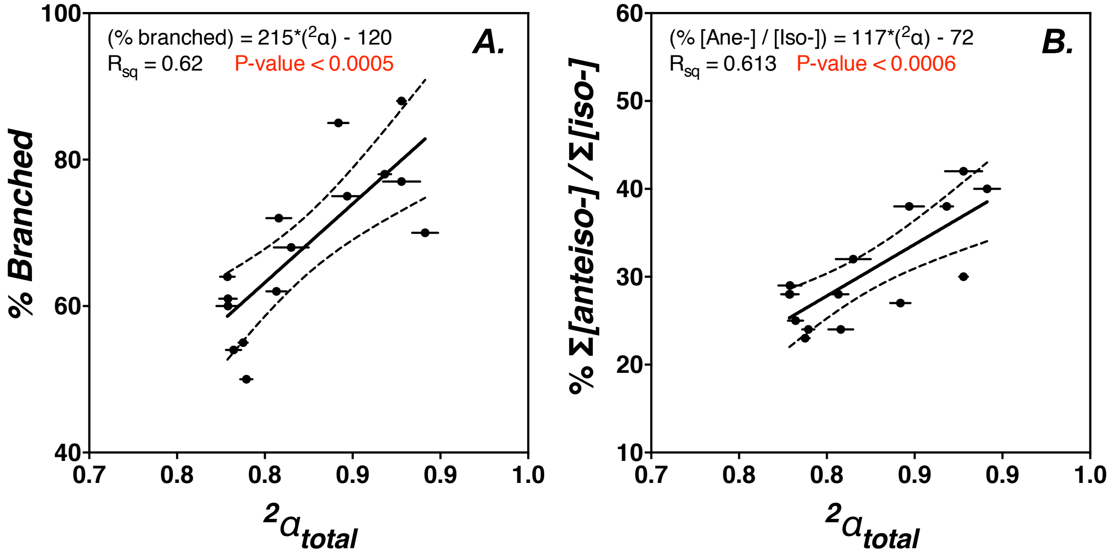

Figure S1. The mass-weighted fractionation between lipids and water versus the proportion of (A) branched fatty acids, (B) the ratio of anteiso- to iso- branched fatty acids.

Figure S2.

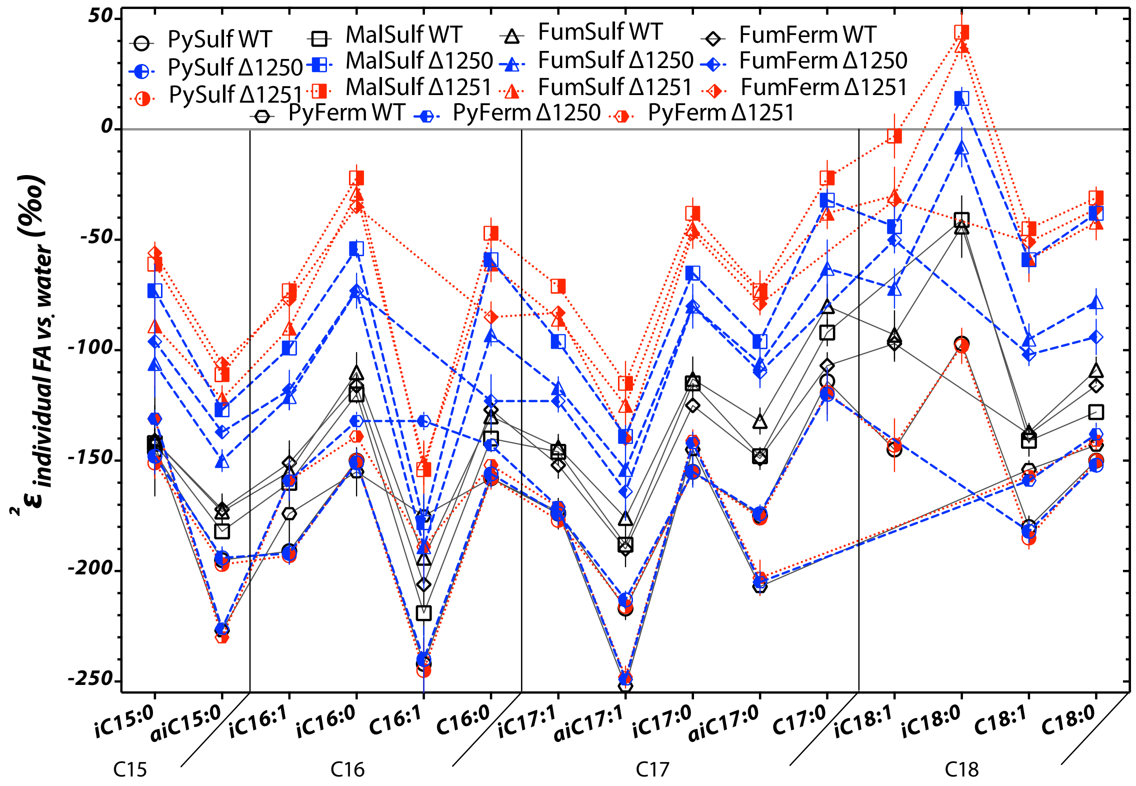

Figure S2. Hydrogen isotope values for individual fatty acids relative to medium water. These are the same values as in Figure 5, but here plotted by compound (x-axis) and legend-coded by experiment. Each symbol represents the mean of biological replicates (N = 2, and technical replication $n_{avg.}$ = 3, range 1 to 6), with SEM.

Figure S3.

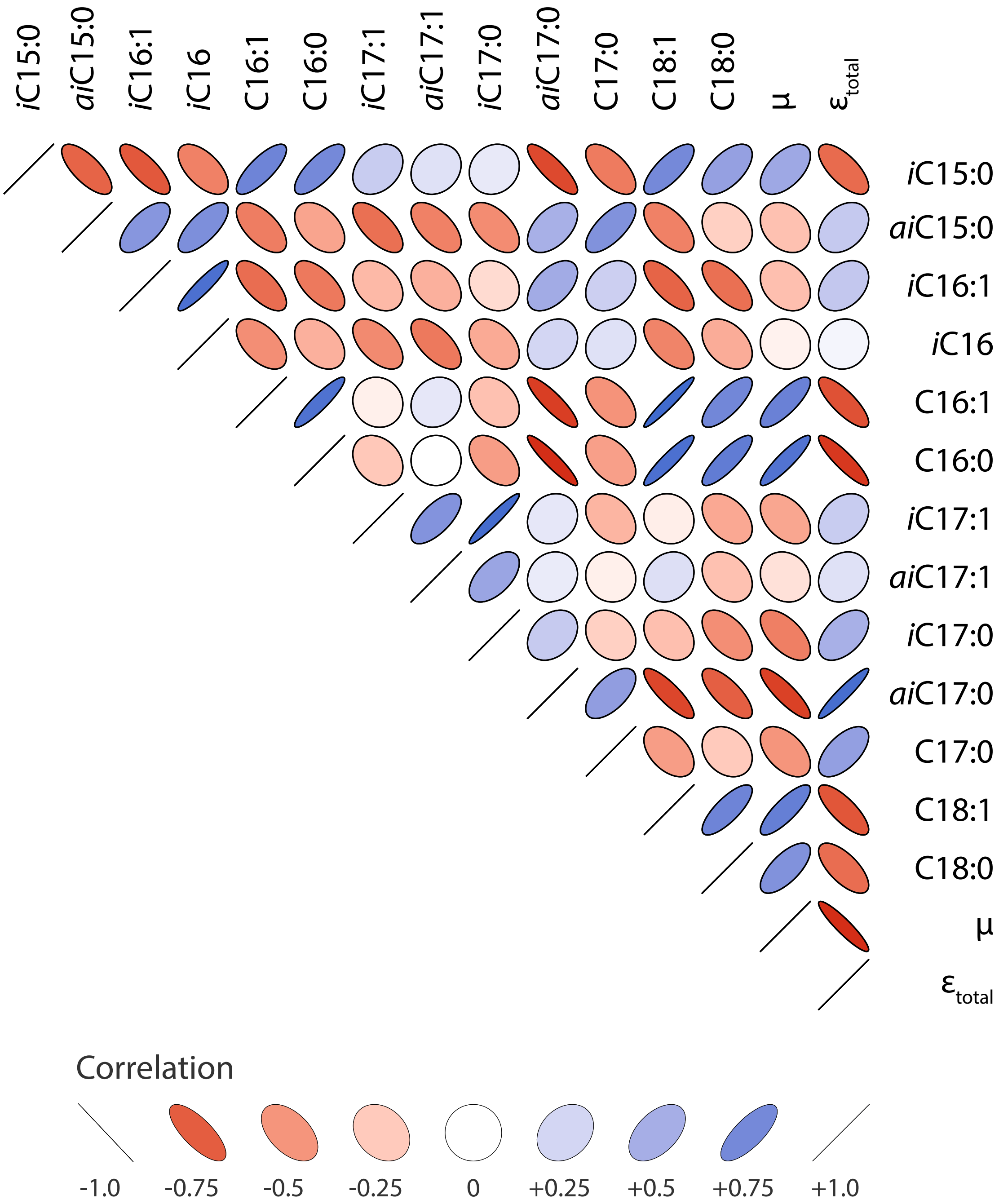

Figure S3. Representation of Pearson correlation indices for each pair of variables. Width of ellipses indicates the strength of the correlation, with narrow ellipses indicating a strong correlation and circles indicating no correlation. Darker blues are stronger positive correlations, darker reds are stronger negative correlations, with white indicating no correlation.

Table S1. FAME identifications based on mass spectra and retention times. For most double bonds, position was determined by DMDS addition.

RI	shorthand	M^+	Free fatty acid
1687	i-C14:0	242	12-methyl tridecanoic acid
1790	i-C15:0	256	13-methyl tetradecanic acid
1798	a-C15:0	256	12-methyl tetradecanoic acid
1871	i-C16:1Δ9	268	14-methyl pentadec-9-enoic acid
1892	i-C16:0	270	14-methyl pentadecanoic acid
1905	C16:1Δ9	268	hexadec-9-enoic acid
1928	C16:0	270	hexadecanoic acid
1967	i-C17:1Δ9	282	15-methyl hexadec-9-enoic acid
1976	a-C17:1Δ9	282	14-methyl hexadec-9-enoic acid
1991	i-C17:0	284	15-methyl hexadecanoic acid
2000	a-C17:0	284	14-methyl hexadecanoic acid
2004	C17:1	282	heptadecenoic acid
2010	C17:1	282	heptadecenoic acid
2027	C17:0	284	heptadecanoic acid
2070	i-C18:1Δ11	296	16-methyl heptadec-11-enoic acid
2090	i-C18:0	298	16-methyl heptadecanoic acid
2108	C18:1Δ11	296	octadec-11-enoic acid
2132	C18:0	298	octadecanoic acid
2738	C24:0	382	tetracosanoic acid (standard)

Table S2. Major mechanisms of NADPH production relevant to *D. alaskensis* G20.

Enzyme	EC number	G20 locus	name
G6PDH	1.1.1.49	Dde_3471	glucose-6-phosphate 1-dehydrogenase
6PGDH	1.1.1.44	Dde_3470	6-phosphogluconate dehydrogenase
IDH	1.1.1.42	Dde_3476	Isocitrate dehydrogenase
ME	1.1.1.40	Dde_1253	malic enzyme
GAPN	1.2.1.9	nd	non-phosphorylating glyceraldehyde 3-phosphate dehydrogenase
NADP+-GAPDH	1.2.1.13	Dde_2342, Dde_3736	Glyceraldehyde-3-phosphate dehydrogenase, type I
GDHs	1.1.1.47, 1.1,1,119	nd	glucose dehydrogenase
STH	1.6.1.1	nd	energy-independent soluble transhydrogenase
H+-TH	1.6.1.2	nd	energy-dependent or proton-translocating,membrane-bound transhydrogenase
FNR	1.18.1.2	Dde_3636, Dde_1251	ferredoxin-NADP reductase
SH	1.12.1.3	Dde_1212	soluble hydrogenase
NADK	2.7.1.23	Dde_2618	ATP-NAD/AcoX kinase
PDR	1.8.1.8	Dde_1301	Protein-disulfide reductase
NfnAB1	1.6.1.4	Dde_3635, Dde_3636	electron-bifurcating transhydrogenase
NfnAB2	1.6.1.4	Dde_1250, Dde1251	electron-bifurcating transhydrogenase

most of these reactions are derived from Table 1 in Spaans et al. (2015) Front. Microbiol.*
